# Modern Sociology

**Published:** 1902-05-17

**Authors:** 


### Modern Sociology.

### ILLS TO WHICH FLESH IS NOT HEIR.

Even people who do not give to sturdy beggars—those able-bodied tramps whose life is one perpetual tour—cannot always resist the petition of those who are maimed or sickly in appearance. Vaguely we know that some of these may be impostors, but we are ready to chance giving to an impostor now and again, rather than that we should deal harshly with a real unfortunate. There is, so far as we know, no English book which shows just how beggars exploit real and pretended infirmities, but M. Louis Paulian has written a detailed account of the ruses of the beggars of Paris, and it is likely that English beggars follow much the same methods.

To begin with real ailments:—M. Paulian had a beggar friend who once expressed envy of a companion who had a rupture and varicose veins. These do not seem desirable characteristics of this fleshly tabernacle; but in the hospitals truss bandages are given gratuitously to the ruptured and elastic stockings to those who have varicose veins. The beggar having used his misfortunes to obtain these helps, at once proceeds to sell them. A second-hand dealer gives 5 francs for the stockings, and 4 francs for the truss bandages. Should the sufferer from these diseases die, or take up another line of mendicancy—when perhaps his face has become too familiar to those who distributed the surgical aids—he can always sell the certificate which shows what disease he is suffering from to another beggar for two francs. It is capital which only wants judicious use to bring in a steady small income to its possessor. Orthopædic apparatus are equally valuable. M. Paulian tells of one man who sold, second-hand, no fewer than six jointed arms in the course of a year. Of course these are sold cheap. A beggar receives a mechanical arm or leg which is worth fifty or sixty francs and sells it for ten or twelve. Even tickets for medicated baths are sold. The beggar does not want a bath any more than he wants an artificial leg when Providence has given him a good asset in the very absence of one of his members. But who buys these things? "Really sick people, the modest poor," says M. Paulian, "the wretched ones in faded frock-coats" who have too much pride or are too feeble to stand in the "queue" at the hospitals, and rather buy what they need at a reduction from the beggar who has obtained them for nothing. So the ticket, the bandages, the surgical appliances, even the cod-liver oil and quinine wine—given to a beggar, who himself, or whose employer, much prefers brandy or absinthe—really come to help the needy in the end. The pity of it is the intervention of the unworthy middleman who makes a living out of the charity he does not deserve.

Some beggars, not content with the misfortunes with which fate has blessed them, simulate another. M. Paulian has noticed that a one-armed man almost invariably seems also to have lost a leg. Also that he usually wears a blouse. If you could investigate the interior of that roomy blouse, you would find the missing leg ingeniously tucked up in it. The beggar sits upon this leg, and thrusts out the other very straight, as if to show aggressively its solitariness. People give him the alms he craves without looking too closely at the details of his appearance. It is the people who don't look who give most freely. In fact, some beggars trust almost audaciously to the lack of intelligence of the charitable. M. Paulian tells the tale of a man, to all appearance an old sailor, whom he saw at a village fair. He was seated on a chair, with a thick rug wrapped round his legs; on his knee was a large picture representing a naval battle. "Ladies and gentlemen," said the old salt, "this is the work of a poor paralytic, who was wounded in defending his country in a great naval engagement." One evening M. Paulian saw this man walking briskly before him, with the naval battlepiece on his back, covered with the rug. He taxed him with being a humbug, and pretending to be paralysed when he was perfectly strong. "Not at all," was the audacious reply. "I never said I was paralysed. I only say: This is the work of a poor paralytic, wounded in defending his country in a great naval engagement. The paralytic is the artist who made the picture. I only show it." This is more elaborate than the epileptic dodge, which requires only the sucking of a bit of soap and a few contortions, and, of course, a sympathetic crowd; but, on the other hand, it can be played more continuously. The drowning trick requires a confederate to make it successful—that is to render it certain that the drowning will be unsuccessful. The drama runs thus:—A hungry-looking, frenzied man throws himself into the river. A sturdy workman flings himself in after him, and with difficulty brings him to land. The suicide then reproaches his rescuer. He is starving, he says; he has neither money nor work. Why was he not left to die. Then the rescuer pulls a sixpence out of his pocket. "That's all I have, but you are welcome to it. I can do without my dinner for once in a way." The sympathetic audience which by this time is sure to have gathered presses its alms on both saviour and saved, arranges that the former shall take the latter home, and sees both into a cab. And off they go—to some public house with which both are familiar, and there the wet clothes are changed for dry ones, and the confederates proceed to enjoy the spoil.

An artist in her way was the woman who, in an omnibus filled chiefly with ladies, began to show all the signs of immediate *accouchement*. She has a pitiful tale—she is starving, has eaten nothing since the previous day, and was going to look for work when—a gasp and a stifled cry break off the narrative. The sympathetic fellow-passengers give her money, stop the omnibus, and send her home in a cab. It is a paying game. Unfortunately the accomplished actress tried the trick again too soon. Within a week she gave another performance at the door of a rich house, but was recognised by one of her former audience, who, now perceiving the trick, threatened her with the police office. The effect was magical. In an instant the suffering woman was on her feet, had pushed the cardboard protuberance which simulated the appearance of an advanced pregnancy behind her, and was off at full speed. That this trick is not unknown on this side of the Channel the present writer can testify. In the early days of the war, when charity was as yet ready and unorganised, I was mulcted in a silver coin and some old baby-linen by a woman who claimed to be a soldier's wife. Several of my neighbours were also moved to practical pity. Unfortunately I commended the woman to the care of the district nurse, who found out not only that there was no soldier husband in the case, but that the expected child was equally mythical. But for one occasion on which the trick is found out, in how many is it successful?

Imposture of this sort is found in every country where wealth and poverty exist side by side in striking contrast. To the wealthy it is far easier to give than to investigate, and beggars are not exclusively to blame for laying traps to catch such inconsidered charity.

